# The use of artificial intelligence and machine learning monitoring to safely administer a fluid-restrictive goal-directed treatment protocol to minimize the risk of transfusion during major spine surgery of a Jehovah’s Witness: a case report

**DOI:** 10.1186/s13256-022-03653-8

**Published:** 2022-11-12

**Authors:** Sara Denn, Emmanuel Schneck, Fidaa Jablawi, Michael Bender, Götz Schmidt, Marit Habicher, Eberhard Uhl, Michael Sander

**Affiliations:** 1 Department of Anesthesiology, Operative Intensive Care Medicine and Pain Therapy, Justus Liebig University of Giessen, Rudolf-Buchheim-Street 7, 35392 Giessen, Germany; 2 Department of Neurosurgery, Justus Liebig University of Giessen, Giessen, Germany

**Keywords:** Jehovah’s Witnesses, Blood transfusion, Hemodynamic monitoring, Case report, Hypotension, Hemodilution

## Abstract

**Background:**

The Hypotension Prediction Index (HPI) displays an innovative monitoring tool which predicts intraoperative hypotension before its onset.

**Case presentation:**

We report the case of an 84-year-old Caucasian woman undergoing major spinal surgery with no possibility for the transfer of blood products given her status as a Jehovah’s Witness. The hemodynamic treatment algorithm we employed was based on HPI and resulted in a high degree of hemodynamic stability during the surgical procedure. Further, the patient was not at risk for either hypo- or hypervolemia, conditions which might have caused dilution anemia. By using HPI as a tool for patient blood management, it was possible to reduce the incidence of intraoperative hypotension to a minimum.

**Conclusions:**

In sum, this HPI-based treatment algorithm represents a useful application for the treatment of complex anesthesia and perioperative patient blood management. It is a simple but powerful extension of standard monitoring for the prevention of intraoperative hypotension.

**Supplementary Information:**

The online version contains supplementary material available at https://doi.org/10.1186/s13256-022-03653-8.

## Background

Rarely is major surgery performed where the transfusion of blood products is not possible. But even under these circumstances, patients should not be deterred from receiving surgical intervention and treatment. Here, we present a case in which a goal-directed therapy based on machine learning hemodynamic monitoring provided a safe framework for conducting invasive spinal surgery in an older patient, who, as a committed Jehovah’s Witness (JW), refused all blood products. For this reason, patient blood management (PBM) played a pivotal role in perioperative management. Of note, pre-existing conditions and advanced age posed an elevated risk to the patient for intraoperative hypotension (IOH). Since IOH is a commonly encountered problem during anaesthesia and is associated with increased mortality and morbidity [1–5], it is vital to stabilize cardiopulmonary function, even during phases of aggravated blood loss.

## Case presentation

The patient gave her written consent to the anonymous description and publication of her case report.

We present the case of an 84-year-old Caucasian woman suffering from a progressive impression fracture of the 11th thoracic vertebral body and severe pain of the left flank of several weeks’ duration. Prior to the patient’s current hospitalization, as reported here, a decompression and stabilization of the lumbar and thoracic spine had been performed 2 months before in a different neurosurgical clinic. In view of the clinical results and continuing severe pain, an extension of the preexisting spinal fusion from the 4th lumbar vertebral body to the second sacral vertebral body, including a cage implantation, was recommended.

The patient presented multiple preexisting conditions, including high blood pressure, diabetes mellitus, diabetic polyneuropathy, moderate valvular aortic stenosis, and restless legs syndrome. Further, the patient introduced herself as a member of the community of JWs. For this reason, she was transferred to the local department of anesthesiology to discuss blood-saving techniques as well as the risks accompanying the procedure, as she was not willing to receive any form of blood transfusion.

After the patient’s decision to undergo spinal fusion surgery, an interdisciplinary plan based on the three pillars of PBM was made to provide maximum security for the patient’s outcome.

### Presurgical assessment

Prior to surgery, the patient signed an informed consent document and was asked to decide which types of blood products would be compatible with her beliefs (Additional file 1: Table S1). Initially, the patient’s hemoglobin (Hb) level was 11.5 g/dl, which could be raised to 12.6 g/dl for the day of surgery by substituting iron-II-glycin-sulfate (100 mg/d).

Additionally, blood sampling was reduced to a minimum, and the surgical procedure was thoroughly and interdisciplinarily planned. Alongside with cell savage techniques, anesthetic management focused on the third pillar of PBM, aiming for cardiopulmonary optimization by using a machine learning monitoring tool and a hemodynamic goal-directed therapy (GDT) protocol. The Hypotension Prediction Index (HPI) was established by Edwards Lifesciences (Irvine, California, USA) and is certified in the United States and Europe for hemodynamic monitoring. It aims to predict phases of intraoperative hypotension (IOH, defined as MAP < 65 mmHg for at least one minute) up to 15 min before their onset. The scale ranges from 0 to 100%, indicating the likelihood of and remaining time to pending IOH [6]. Our hypothesis was that unnecessary volume expansion could be avoided by using the HPI-based GDT because additional fluids would only be given when the patient would be volume responsive and simultaneously be at risk for IOH. This approach should therefore prevent unnecessary hemodilution.

### Induction of anesthesia, hemodynamic, and intraoperative management

The patient received a standard induction of anesthesia using sufentanil (0.52 μg/kg), propofol (1.67 mg/kg), and cis-Atracurium (0.21 mg/kg). The maintenance of anesthesia was performed as total intravenous anesthesia using propofol (6–12 mg/kg/h) as continuous infusion and sufentanil (0.1–0.3 μg/kg) as intermittent bolus injection. The total duration of surgery was 425 min. To compensate for blood loss, a mechanical autotransfusion system (CellSaver®, Haemonetics Corporation, Boston, USA) was used during the whole course of surgery.

**Fig. 1:**
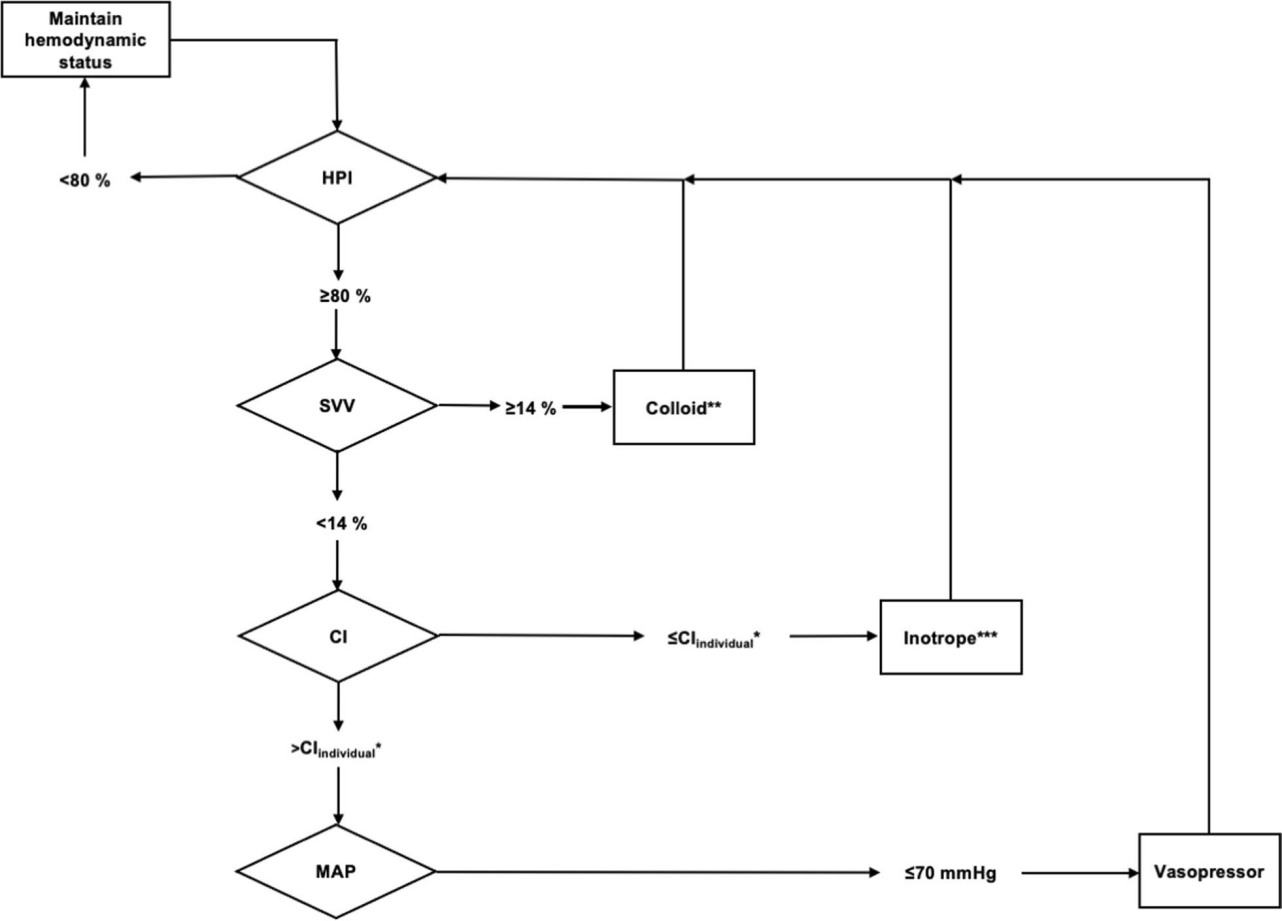
Hypotension Prediction Index-based hemodynamic treatment algorithm*. *CI: Cardiac Index; HPI: Hypotension Prediction Index; MAP: Mean Arterial Pressure; SVV: Stroke Volume Variation

For hemodynamic management, an HPI-based GDT-algorithm was used (Fig. 1). Since unnecessary hemodilution, also in GDT-treated patients, would lead to an unfavorable outcome, especially in the presented patient, we chose a more fluid-restrictive GDT [7, 8]. First, before the induction of anesthesia, the baseline cardiac index (CI) as well as the stroke volume were determined by implementing an arterial line in the awake patient. If HPI was raised over 70%, hemodynamic intervention was performed according to the algorithm. More specifically, if stroke volume variation (SVV) increased over 14%, a colloid bolus injection using 100 ml of gelatin infusion (Gelafundine®, B. Braun Germany) was applied over 5 min. If HPI did not decrease, the injection was repeated until HPI declined < 80% or SVV < 14%, whichever occurred first. If HPI persistently leveled > 80% despite a successful fluid challenge (defined as SVV < 14%), CI was raised when it was below the baseline value with dobutamine (5 μg/kg/min, half dose if heart rate was raised > 100 bpm). If still no reduction of HPI could be achieved after successful fluid challenge and CI optimization, noradrenaline or theodrenaline/cafedrine (Akrinor®) was used to maintain a MAP > 70 mmHg.

By adhering to the GDT algorithm, a sufficient hemodynamic stability was achieved throughout the surgery. The patient received a total of 2500 ml colloid infusion, maximum rates of 0.13 µg/kg/min of noradrenaline, 0.5 ml of theodrenaline/cafedrine, and no further cardiac support medication. The patient never showed signs of low pH or negative BE despite a blood loss of 2.4 L (Table 1). It was possible to reinfuse 1007 ml of the patient’s own blood that was saved by the autotransfusion system. To prevent hypofibrinolytic bleeding, tranexamic acid was infused intraoperatively, starting with a bolus of 20 mg/kg/h for 1 hour and continuing with an infusion of 4 mg/kg/h for the remaining time of surgery. Once bleeding started to intensify, point-of-care thrombelastography (ROTEM®, TEM Innovations, Munich, Germany) was used to detect coagulatory alterations, which resulted in the substitution of 3 g fibrinogen.

**Table 1:** Results of the blood gas analyses

	Before surgery	1 h after beginning of surgery	4 h into surgery	End of surgery	Admission at ICU	12 h postop	24 h postop	48 h postop	Discharge from ICU
Hb (g/dl)	11.8	12.0	9.5	6.72	5.9	4.1	3.4	3.8	6.6
Hct (%)	36.4	36.8	29.5	20.9	18.5	13.3	11.1	12.3	20.7
pH	7.5	7.39	7.34	7.36	7.64	7.32	7.54	7.58	7.46
BE (mmol/L)	4.2	0.4	0.8	− 0.2	4.6	− 8.6	6.0	9.7	− 3.2
SpO_2_ (%)	97.8	98.5	98.4	98.3	97.9	99.7	99.8	99.8	97.7
Lactate (mmol/L)	0.65	0.84	0.83	1.64	1.8	1.3	2.0	2.1	0.7
pO_2_ (mmHg)	391	273	250	245	246	146	155	131	87
pCO_2_ (mmHg)	34	41.2	49.5	44.6	23.8	33.2	33.8	36.6	27.9

BE: Base Excess; Hb: hemoglobin; Hct: hematocrit

### Follow-up and outcomes

Upon admission to the neurosurgical intensive care unit, initial hemoglobin leveled at 5.6 g/dl and hematocrit at 0.15%, respectively, with a total measurement of erythrocytes at 1.8 tera/L. Even with anemia being present, the patient did not show elevated lactate levels, tachycardia, or arrhythmia. Coagulatory analysis showed a prothrombin time of 49%, an international normalized ratio of 1.5, and antithrombin III of 40% (of normal range). The patient immediately received 3000 International Units (I.U.) of prothrombin complex concentrate and 4000 I.U. on the first postoperative day. Further, 2000 I.U. of AT-III as well as 800 ml of 20% albumin, 100 mg of iron-III-hydroxide-Infusion, and 20,000 I.U. of erythropoietin (EPO) were substituted within the first hours after surgery and again on the first, second, and tenth days after surgery. Initially, a higher dosage (0.208 µg/kg/h) of norepinephrine was needed, but this could be reduced within 4 hours after the patient’s volume status was balanced.

## Discussion

To our knowledge, this is the first time that a machine learning algorithm was used to safely utilize a fluid-restrictive GDT treatment protocol in a JW patient to minimize the risk of transfusion and to still guarantee hemodynamic optimization.

There are various ways to practice pre-, intra-, and postoperative PBM during high-risk surgical procedures. However, the inability to use blood products presents a uniquely challenging situation. In this case, the patient maintained a very strict view on the usage of blood products, leaving the anesthesiologist with a limited scope of therapeutic actions at their disposal. Due to the extensive nature of the surgery, it was necessary to focus not only on Hb- and iron compensation beforehand, but also on techniques that could optimize cardiopulmonary function respective to the corresponding oxygen delivery. Furthermore, it was vital to maintain a balanced hemodynamic status due to the patient’s age and various preexisting conditions. For this reason, the attending anesthesiologist chose to use an HPI-based hemodynamic GDT in order to treat hemodynamic instability and hypotensive events before their occurrence. This treatment approach led to a high degree of hemodynamic stability without putting the patient at risk of a too-liberal fluid therapy with consequent hemodilution. More specifically, in many GDT algorithms, fluid optimization is used by maximizing stroke volume, but the downside of this approach might be a fluid strategy that is too liberal: as with the last fluid bolus that does not significantly increase stroke volume, indicating that the patient is at top of its individual starling curve, additional fluid is infused, which actually decreases oxygen delivery by not further increasing cardiac output but leading to hemodilution. Such an approach might be especially detrimental in patients where blood transfusion is not an option. Therefore, our approach was to use the HPI monitoring protocol that would only indicate the need for hemodynamic optimization and fluid expansion in the case of imminent IOH, and would therefore offer a more fluid-restrictive strategy, minimizing the risk of hemodilution in this vulnerable patient.

**Fig. 2:**
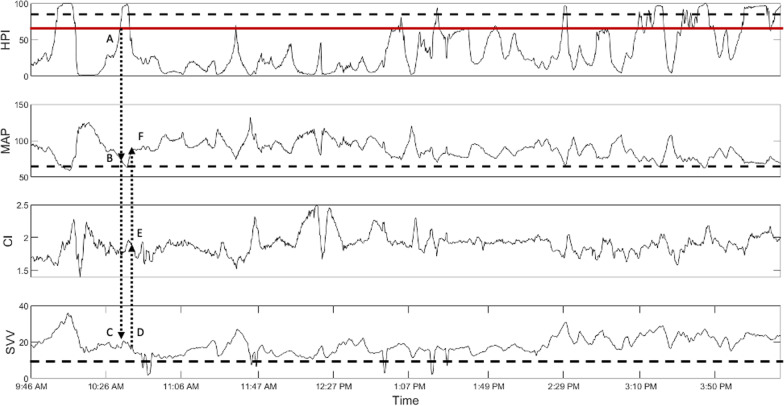
Time line of the Hypotension Prediction Index, mean arterial pressure, cardiac index, and stroke volume variation. The dotted line indicates an HPI of 90%, respectively the threshold MAP of 65 mmHg and SVV of 14%. The red line shows the the interventional HPI-cutoff of 70%. The dotted arrows demonstrate an example of an HPI-triggered intervention: The HPI (*A*) indicated an episode of hypotension prior to its onset (*B*) which was associated to an increase of the SVV (*C*). As a consequence a colloid bolus was administered lowering the SVV (*D*), simultaneously increasing the CI (*E*) and finally restoring the MAP (*F*). CI: Cardiac Index; HPI: Hypotension Prediction Index; MAP: Mean Arterial Pressure; SVV: Stroke Volume Variation

HPI monitoring has been evaluated in several studies as regards the primary goal of reducing IOH, since it continues to present a common problem during anesthesia and is associated with increased mortality and morbidity [1–5]. For example, mortality 1 year after anesthesia exposure is raised by 3.6% for every minute the systolic arterial blood pressure (SAP) remained below 80 mmHg. Furthermore, the mortality risk is 1.4 times higher when the mean arterial blood pressure (MAP) decreased under 55 mmHg for more than 10 min [2, 8]. Davies* et al*. outline a significant reduction of IOH by using an HPI-based GDT protocol [9]. Recently, our own study on patients undergoing hip replacement surgery showed that the use of HPI can significantly reduce the incidence and duration of IOH [6]. Further, Wijnberge* et al*. described the occurrence of less IOH in HPI-guided therapy compared with standard care [10]. Also, in the presented case, the duration of IOH (defined as MAP < 65 mmHg) could be reduced to just 8 min by strict adherence to the HPI-based GDT (Fig. 2). The data evaluation showed a total of 55 min with an HPI > 70% (of 425 min), resulting in 21 interventions that included colloid and noradrenaline infusions. The patient’s mean MAP over the total course of surgery was 88 mmHg. Further, cardiac function was preserved over the course of surgery despite the extended blood loss (2.4 L, Hb drop of 11.8 to 6.7 g/dl), and the patient never suffered any form of acidosis (Table 1).

PBM plays a pivotal role in high-risk bleeding surgeries, especially for someone who is a JW. The optimization of red blood cell production can easily be managed using EPO or iron substitutes before surgery. Also, surgical techniques have massively improved the provision of minimally invasive methods to help reduce blood loss, as has the use of tranexamic acid. However, in the opinion of the authors, hemodynamic optimization must be highlighted more in the context of PBM, since it not only reduces IOH and accompanying risks, but it can also keep patients hemodynamically stable, maximizing the tolerance of low Hb levels by directing individual volume demand. Most studies on the perioperative management of a JW patient concentrate on oral iron substitution and intraoperative normovolemic hemodilution, respectively, as well as the use of autotransfusion techniques. For example, without focusing on hemodynamic optimization, McCartney* et al*. were able to show that the abovementioned therapeutic actions were already associated with an improved clinical outcome after cardiac surgery of JWs [11–13]. Also, several case reports have supported preoperative EPO administration and blood-saving surgical techniques [14]. Today, even though the clinical implementation of these strategies is commonly applicable, it should also be focused on individual hemodynamic stabilization.

## Conclusions

In sum, our approach demonstrates how the use of an HPI-guided GDT in a transfusion-limited surgical context led to a high degree of hemodynamic stability while reducing the risk for dilutional anemia, and thus represents an extension of PBM both in general and for the JW community in particular.

## Supplementary Information

**Additional file 1: Table S1.** Patient´s consent on the application of different blood products.

## Data Availability

Data sharing is not applicable to this article as no datasets were generated or analysed during the current study.

## Abbreviations

AT-III: Antithrombin III
BE: Base excess
CI: Cardiac Index
EPO: Erythropoietin
GDT: Goal-directed therapy
Hb: Hemoglobin
HPI: Hypotension Prediction Index
IOH: Intraoperative hypotension
I.U.: International units
JW: Jehovah’s Witness
MAP: Mean arterial blood pressure
PBM: Patient blood management
SAP: Systolic arterial blood pressure
SVV: Stroke volume variation
