# Supplementary material for: The use of artificial intelligence and machine learning monitoring to safely administer a fluid-restrictive goal-directed treatment protocol to minimize the risk of transfusion during major spine surgery of a Jehovah’s Witness: a case report
Source: J Med Case Rep. 2022 Nov 12;16:412. doi: 10.1186/s13256-022-03653-8 (PMC9652964; doi:10.1186/s13256-022-03653-8)
Supplement: Supplementary file 1 — Additional file 1: Table S1. Patient´s consent on the application of different blood products. [file 13256_2022_3653_MOESM1_ESM.docx]

**Additional file**

**Table S1**

| **Blood and blood derivates** | **yes** | **no** |
| --- | --- | --- |
| Erythropoietin | ✓ |  |
| Desmopressin | ✓ |  |
| Antifibrinolytics | ✓ |  |
| Autologous transfusion |  | ✓ |
| Mechanic autotransfusion | ✓ |  |
| Renal replacement therapy | ✓ |  |
| Extracorporeal membrane oxygenation | ✓ |  |
| Controlled hypotension | ✓ |  |
| Erythrocyte concentrates |  | ✓ |
| Platelet concentrates |  | ✓ |
| Fresh Frozen Plasma |  | ✓ |
| Coagulation factors  (e.g., Fibrinogen, Antithrombin III) | ✓ |  |
| Immunoglobulins | ✓ |  |
| Albumin | ✓ |  |
| Interferons | ✓ |  |
| Recombinant Factor VIIa (NovoSeven^®^) | ✓ |  |

**Table S1:** Patient´s consent on the application of different blood products.
